# Gender-Specific Bile Acid Profiles in Non-Alcoholic Fatty Liver Disease

**DOI:** 10.3390/nu16020250

**Published:** 2024-01-13

**Authors:** Julia Fitzinger, Giovanny Rodriguez-Blanco, Markus Herrmann, Andrea Borenich, Rudolf Stauber, Elmar Aigner, Harald Mangge

**Affiliations:** 1 Clinical Institute of Medical and Chemical Laboratory Diagnostics, Medical University of Graz, 8036 Graz, Austria; julia.fitzinger@stud.medunigraz.at (J.F.); markus.herrmann@medunigraz.at (M.H.); 2 Institute for Medical Informatics, Statistics and Documentation, Medical University of Graz, 8036 Graz, Austria; andrea.borenich@medunigraz.at; 3 Division of Gastroenterology and Hepatology, Medical University of Graz, 8036 Graz, Austria; rudolf.stauber@medunigraz.at; 4 First Department of Medicine, University Clinic Salzburg, Paracelsus Medical University Salzburg, 5020 Salzburg, Austria; e.aigner@salk.at

**Keywords:** bile acid profiles, NAFLD, ALD, gender differences

## Abstract

Background: Non-alcoholic fatty liver disease (NAFLD) is increasing worldwide. A main cause is the obesogenic, so-called Western lifestyle. NAFLD follows a long, unperceived course, and ends potentially fatally. Early diagnosis of aggressive subtypes saves lives. So far, non-invasive means of detection are limited. A better understanding of the pathogenic interplay among insulin resistance, immune inflammation, microbiome, and genetic background is important. Metabolomics may give insight into these interlaced processes. Methods: In this study, we measured bile acids (BA) in the plasma of adult NAFLD and alcohol-associated liver disease (ALD) patients and healthy controls with targeted mass spectrometry. We focused on gender-related bile acid production pathology in NAFLD and ALD. Results: Compared to healthy controls, women with NAFLD had significantly higher concentrations of total BA, total primary BA, total cholic (CA), total chenodeoxycholic (CDCA), total glycine-conjugated, and total non-12-a-OH BA. Concerning subtypes, glycocholic (GCA) and glycochenodeoxycholic (GCDCA), BA were elevated in women with NAFLD. In contrast, men with NAFLD had no significantly altered total BA fractions. However, the subtypes GCA, glycodeoxycholic (GDCA), glycolithocholic (GLCA), lithocholic (LCA), taurolithocholic (TLCA), and tauroursodeoxycholic acid (TUDCA) were elevated, while CA was significantly decreased. In NAFLD, except ursodeoxycholic acid (UDC), all total BA correlated significantly positively in both sexes with the ELF score, while in ALD, only males showed significant correlations exceptive for total UDC BA. In NAFLD, total BA, total primary BA, total secondary BA, total free secondary BA, total CA, total CDCA, total taurine conjugated, total glycine conjugated, total 12-a-OH, and total non-12-a-OH were significantly higher in cases of a high enhanced liver fibrosis (ELF) score above 9.8. In ALD, total UDC was additionally elevated. Between NAFLD with and without NASH, we found no significant differences. Conclusion: Our data show gender-specific bile acid profiles in NAFLD and markedly different BA patterns in ALD. Women with NAFLD had more severe cholestasis. Men may better compensate fat storage-driven bile acid dynamics, indicated by higher levels of taurine-conjugated BA, which associate with beneficial metabolic functions.

## 1. Introduction

Non-alcoholic fatty liver disease (NAFLD) is increasing worldwide in young people and adults [1]. Currently, around 30% of the general world population shows increased amounts of fat in the liver, an early sign of metabolic imbalance. Up to 70% of obese people or those with T2DM have NAFLD [2]. Non-alcoholic fatty liver disease is a leading cause of liver-related hospitalisations and deaths [3]. Non-alcoholic fatty liver disease shows, in many cases, a strong metabolic component; thus, a renaming as “metabolic dysfunction-associated steatotic liver disease” (MASLD) has been discussed [4,5,6,7] to draw a clearer line to other potential non-alcoholic causes of steatosis. Abdominal obesity, type 2 diabetes mellitus, dyslipidaemia, increased blood pressure, and chronic low-grade inflammation are important drivers of NAFLD. Moreover, advanced age, male sex, ethnicity, environmental factors, lifestyle, and genetic variations are involved [3]. Increased childhood obesity leads to earlier onset of NAFLD, and there are higher rates of progressive liver disease in the elderly [3]. Recently, dysbiosis of gut microbiota was connected with an accelerated progression of NAFLD [3].

Earlier phases of NAFLD include simple hepatic steatosis (NAFL, HS) and non-alcoholic steatohepatitis (NASH). This process progresses or remains stable over a long period. Fibrosis, cirrhosis, and hepatocellular carcinoma (HCC) can develop in the course of disease progression. About 20% of NASH patients show rapid progression to advanced fibrosis within a few years. Of these, about 20% progress to cirrhosis, of which after two years, 20% present hepatic decompensation [3].

Despite its close connection to obesity, the significance of NAFLD in lean patients may be underestimated. According to a Chinese study, the risk of metabolic syndrome and hypertension in lean NAFLD patients was comparable to obese NAFLD patients, if the visceral adipose index (VAI) was significantly elevated [8]. Lean NAFLD patients show a higher risk rate to be a carrier of the PNPLA3 allele compared to lean controls [8,9,10]. PNPLA3 rs738409 polymorphism is associated already with young people with increased liver enzymes [11].

Bile acids (BA) play an important role in the pathogenesis of NAFLD. The synthesis of BA utilises cholesterol and starts in hepatocytes following two main pathways. In the classical pathway, side-chain cleavage follows sterol ring modification; in the alternate pathway, it is reversed. Sufficient functionality of these pathways prevents accumulation of cholesterol in the liver. Among mammals, all BA have a C24- construct, but their structures vary between different species. The circulating BA pool is a mixture of primary and secondary BA. Cholic acid (CA) and chenodeoxycholic acid (CDCA) are human primary BA and are conjugated with the amino acids glycine or taurine by bile acid–CoA synthase (BACS) and bile acid–CoA:amino acid N-acetyltransferase (BAAT) in the liver cells. The conjugated primary BA, glycocholic acid (GCA), taurocholic acid (TCA), glycochenodeoxycholic acid (GCDCA), and taurochenodeoxycholic acid (TCDCA), show an increase in solubility in physiological pH, ionisation, and less passive absorption.

In the distal intestine, bacterial 7α-dehydroxylase transforms the unabsorbed fraction of primary BA to the secondary BA, deoxycholic acid (DCA) and lithocholic acid (LCA). Lithocholic acid is also the product of 7β-dehydroxylation from ursodeoxycholic acid (UDCA). Bile acids are recycled efficiently (95%) by reabsorption through the brush border membrane in the terminal ileum. 

Apart from their direct role in lipid emulsification and solubilisation, BA work as nuclear receptor ligands on the farnesoid X receptor (FXR), vitamin D receptor (VDR) and pregnane X receptor (PXR) and on membranous receptors like the Takeda G-protein-receptor-5 (TGR5) [12,13]. The Farnesoid X receptor is involved in regulating BA synthesis, secretion, and distribution. De novo lipogenesis (DNL) and hepatic VLDL secretion are supressed by activation of FXR. Generally, FXR activation improves metabolic syndrome by lowering blood glucose, improving insulin resistance, and decreasing levels of FFA [14]. In NAFLD patients, studies found that expression of FXR and signalling were decreased [12,15,16].

There also is an interplay between the bile acid pool and microbiota. A changing presence in certain species in NAFLD goes along with a variation in bacterial enzymes and, therefore, a change in conjugation and conversion of BA [17].

Furthermore, obesity associates closely with fatty liver disease [18]. Although sex-specific differences in body fat distribution have been well demonstrated [18], little is known about sex-specific associations between adipose tissue distribution and NAFLD [18]. Bile acid production may also play a role in this context. Thus, one focus of our study was the analysis of sex-related differences in BA production in NAFLD.

Bariatric surgery (BS) improved non-alcoholic fatty liver disease (NAFLD) [19]. Nevertheless, some patients did not respond to BS. Specific changes in gut microbiota and plasma bile acids may contribute to resolving NAFLD in BS patients. Responder patients showed a greater abundance of Bacteroides, Akkermansia, and several species of the Clostridia class, along with a decreased abundance of Actinomycetes/Bifidobacterium and Faecalicatena. After BS, NAFLD resolution associated also with a sustained increase in primary bile acids (particularly non-conjugated), which may result from a reduction in bacterial gut species capable of generating secondary bile acids [19].

Metabolomics may give insight in the alterations of BA pathways, helping to understand the pathophysiology of diseases by measuring metabolites that are part of biological processes in the body [20,21]. In this study, we investigated bile acid profiles of NAFLD patients compared to early-stage alcohol-associated liver disease (ALD) and healthy controls with emphasis on sex-specific differences in BA production in NAFLD.

We hypothesise that more active liver disease may associate with specific bile acid profiles in both non-alcoholic and alcohol-associated fatty liver disease, giving a better insight into the involved pathogenic mechanisms. 

## 2. Material and Methods

### 2.1. Study Design

In total, we analysed 205 patients. A total of 45 NAFLD patients (28 male, 17 female) were enrolled at the Division of Gastroenterology Medical University of Graz, and 103 matched healthy controls (51 male, 52 female) were from the Paracelsus-1000 cohort from the Paracelsus Medical University of Salzburg. Finally, 57 (45 male, 12 female) patients with early-stage ALD undergoing alcohol detoxification therapy were enrolled at the Division of Gastroenterology Medical University of Graz. 

### 2.2. Laboratory Work

After introducing internal standards (d4-DCA, d4-LCA, d4-GLCA, d4-GCDCA, and d4-TDCA, each at a concentration of 0.2 nmol), samples (10 μL) were vigorously mixed for 1 min. To facilitate removal of proteins, we added 400 μL of acetonitrile. The mixture was centrifuged at 3200× *g* for 12 min at room temperature. The supernatant was removed carefully and subsequently evaporated under a flow of nitrogen. The dried samples were reconstituted using 100 μL of mobile phase B and subsequently transferred into vials suitable for auto sampling.

### 2.3. Mass Spectrometry Analyses 

Bile acids were analysed using liquid chromatography high-resolution mass spectrometry (LC-HR-MS). Chromatography of 10 µL of each sample was performed using a Nucleoshell C18 reversed-phase column (Macherey-Nagel, Düren, Germany) for human bile acids. Separation was performed using aqua dest with 1.2% *v*/*v* formic acid and 0.38% *w*/*v* ammonium acetate, and elution was carried out using acetonitrile with 1.3% *v*/*v* formic acid and 0.38% ammonium acetate. Analysis was performed using a Triple Quadrupole mass spectrometer 6500 (Sciex, Waltham, MA, USA) with an ESI ion source in negative ionisation mode. The limit of quantitation of the mass spectrometer was 0.001 µmol/L for all bile acid species. Values below this threshold were not quantitated and excluded from statistical analysis. 

### 2.4. Enhanced Liver Fibrosis (ELF) Test

The enhanced liver fibrosis (ELF) test is a proprietary fibrosis panel based on extracellular matrix proteins containing hyaluronic acid (HA), procollagen-3 *N*-terminal peptide (P3NP), and tissue inhibitor of metalloproteinase-1 (TIMP-1). Serum samples were used to perform the ELF test on an Advia Centaur XP (Siemens Healthcare Diagnostics, Vienna, Austria). For diagnosis of advanced fibrosis, the published ELF score cut-off of 9.8 was applied.

### 2.5. Statistics

Metric data were reported as mean and standard deviation (SD) if normally distributed or median and interquartile range (IQR) if not. Categorical data were summarised as relative and absolute frequencies. Reported percentages always pertain to the number of non-missing answers. As appropriate, comparisons between groups (NAFLD, ALD, controls) were performed using the Mann–Whitney U test or *t*-test. Comparisons between low/moderate and high ELF scores and between patients with NAFLD without and with NASH were conducted using the Mann–Whitney U test, *t*-test, Pearson’s Chi-squared test, or Fisher’s exact test. Correlations were determined using Spearman’s correlation coefficient. For comparisons between groups (NAFLD, ALD, controls), a two-sided *p*-value of 0.0167 was considered statistically significant as the Bonferroni correction was used to adjust for multiple testing; otherwise, a *p*-value of 0.05 was used. All statistical analyses were conducted using R version 4.3.1.

## 3. Results

### 3.1. Table 1 and Table 2 Show Baseline Anthropometric and Clinical Characteristics of Female and Male Participants

The NAFLD group for both genders had the highest BMI, followed by ALD compared to the controls (Table 1 and Table 2). Median levels of fasting glucose were below 100 mg/dL in the alcoholic and control groups and were significantly higher in the NAFLD group (Table 1 and Table 2). 

**Table 1:** Baseline anthropometric and clinical characteristics for female participants.

Characteristic	NAFLD, N = 17 ^1^	Controls, N = 52 ^1^	ALD, N = 12 ^1^	NAFLD vs. Controls ^2^	NAFLD vs. ALD ^2^	Controls vs. ALD ^2^
Age	53 (13)	55 (8)	48 (8)	0.587	0.234	**0.022**
BMI	32.7 (27.3, 38.4)	21.6 (20.5, 23.1)	28.3 (22.4, 31.7)	**<0.001**	0.084	**0.002**
Waist (cm)	111 (100, 118)	78 (73, 82)	NA (NA, NA)	**<0.001**	NA	NA
Fasting Glucose (mg/dL)	110 (95, 143)	87 (84, 90)	89 (85, 91)	**<0.001**	**0.001**	0.404
AST (U/L)	50 (38, 76)	21 (18, 23)	26 (23, 37)	**<0.001**	**0.005**	**0.001**
ALT (U/L)	71 (33, 95)	17 (14, 20)	24 (20, 28)	**<0.001**	**<0.001**	**0.001**
GGT (U/L)	122 (72, 195)	16 (11, 20)	55 (37, 77)	**<0.001**	**0.011**	**<0.001**
Cholinesterase (U/L)	8984 (6936, 9624)	7500 (6588, 8636)	5959 (5037, 7320)	0.096	**0.014**	0.025
Alcaline phosphatase (U/L)	111 (78, 121)	60 (50, 70)	69 (53, 81)	**<0.001**	**0.010**	0.203
Tchol (mg/dL)	217 (190, 254)	210 (189, 228)	209 (197, 230)	0.807	>0.999	0.979
HDL (mg/dL)	45 (39, 53)	84 (69, 93)	60 (48, 74)	**<0.001**	0.086	**0.005**
LDL (mg/dL)	136 (97, 150)	135 (106, 152)	128 (100, 151)	0.561	>0.999	0.681
TG (mg/dL)	122 (100, 170)	32 (28, 40)	102 (60, 123)	**<0.001**	0.073	**<0.001**
Creatinin (mg/dL)	0.78 (0.71, 0.91)	0.75 (0.69, 0.81)	0.73 (0.60, 0.87)	0.132	0.307	0.890
Urea nitrogen (mg/dL)	31 (26, 36)	28 (24, 32)	26 (18, 30)	0.107	0.073	0.222
Uric acid (mg/dL)	5.50 (4.80, 6.00)	3.98 (3.61, 4.50)	4.40 (3.68, 5.10)	**<0.001**	0.035	0.302
Thrombocytes (G/L)	222 (191, 272)	253 (230, 290)	240 (208, 286)	0.066	0.352	0.497
C-reactive protein (mg/dL)	4.8 (2.1, 8.0)	0.1 (0.1, 0.1)	3.8 (1.6, 9.5)	**<0.001**	0.955	**<0.001**
Fib-4 index	1.87 (1.06, 2.42)	1.14 (0.92, 1.30)	1.13 (0.72, 1.43)	**0.016**	0.088	0.904
FLI score	93 (82, 98)	3 (2, 5)	NA (NA, NA)	**<0.001**		
ELF Score	9.24 (8.48, 10.37)	NA (NA, NA)	8.57 (8.25, 9.31)		0.438	
NAFLD Fibrosis Score	−0.57 (−1.48, −0.09)	−2.71 (−3.00, −2.06)	−2.23 (−2.31, −1.15)	**<0.001**	0.088	0.109

^1^ Mean (SD); dedian (IQR). ^2^ The significance level is 0.0167 due to Bonferroni correction (shown in bold *p* values). NA = Not available.

**Table 2:** Baseline anthropometric and clinical characteristics of male participants.

Characteristic	NAFLD, N = 28 ^1^	Controls, N = 51 ^1^	ALD, N = 45 ^1^	NAFLD vs. Controls ^2^	NAFLD vs. ALD ^2^	Controls vs. ALD ^2^
Age	50 (14)	53 (7)	48 (9)	0.397	0.435	0.007
BMI	28.9 (25.9, 30.9)	22.7 (21.8, 24.0)	26.2 (24.2, 29.4)	**<0.001**	0.024	**<0.001**
Waist (cm)	103 (99, 110)	86 (82, 91)	NA (NA, NA)	**<0.001**		
Fasting Glucose (mg/dL)	99 (92, 113)	89 (84, 94)	88 (83, 94)	**<0.001**	**<0.001**	0.964
AST (U/L)	52 (41, 72)	23 (20, 27)	31 (24, 50)	**<0.001**	**0.001**	**<0.001**
ALT (U/L)	82 (57, 143)	20 (18, 23)	37 (25, 58)	**<0.001**	**<0.001**	**<0.001**
GGT (U/L)	115 (77, 282)	20 (16, 25)	121 (47, 292)	**<0.001**	0.371	**<0.001**
Cholinesterase (U/L)	8335 (7690, 9596)	7398 (6602, 8253)	7629 (6374, 8272)	**0.004**	**0.009**	0.849
Alcaline phosphatase (U/L)	70 (62, 81)	54 (47, 68)	67 (60, 87)	**<0.001**	0.921	**<0.001**
Tchol (mg/dL)	186 (175, 222)	193 (173, 218)	188 (150, 214)	0.914	0.578	0.340
HDL (mg/dL)	40 (33, 46)	64 (59, 73)	45 (34, 56)	**<0.001**	0.220	**<0.001**
LDL (mg/dL)	112 (85, 142)	130 (113, 151)	116 (95, 140)	0.048	0.781	0.051
TG (mg/dL)	139 (99, 178)	68 (50, 80)	97 (75, 133)	**<0.001**	0.018	**<0.001**
Creatinin (mg/dL)	0.99 (0.91, 1.04)	0.91 (0.82, 1.01)	0.83 (0.76, 0.96)	0.061	**0.002**	0.024
Urea nitrogen (mg/dL)	33 (30, 40)	29 (25, 33)	22 (18, 28)	**0.001**	**<0.001**	**<0.001**
Uric acid (mg/dL)	6.40 (5.33, 7.30)	5.22 (4.58, 5.77)	5.60 (4.90, 6.30)	**<0.001**	0.064	0.042
Thrombocytes (G/L)	198 (152, 225)	262 (223, 282)	257 (214, 283)	**<0.001**	**<0.001**	>0.999
C-reactive protein (mg/dL)	1.2 (0.7, 3.2)	0.1 (0.0, 0.1)	2.6 (1.3, 4.4)	**<0.001**	**0.008**	**<0.001**
Fib-4 index	1.41 (0.89, 2.87)	1.16 (0.93, 1.38)	1.02 (0.74, 1.36)	0.088	0.045	0.274
FLI score	88 (77, 92)	15 (9, 20)	NA (NA, NA)	**<0.001**		
ELF Score	8.35 (8.20, 9.77)	NA (NA, NA)	9.45 (8.51, 10.03)		0.077	
FiB_Score	−1.11 (−2.63, 0.10)	−2.16 (−3.20, −1.60)	−2.73 (−3.32, −1.94)	0.033	**0.015**	0.356

^1^ Mean (SD); median (IQR). ^2^ The significance level is 0.0167 due to Bonferroni correction (shown in bold *p* values). NA = Not available.

Aminotransferases (AST, ALT) and cholinesterase were significantly higher in NAFLD than in ALD and controls. GGT was significantly elevated in NAFLD and ALD compared to controls (Table 1 and Table 2). In ALD, males had higher GGT than females (121 (47, 292) vs. 55 (37, 77), *p* = 0.044). Alkaline phosphatase was elevated in NAFLD compared to controls, and in males, they were significantly higher than in ALD (Table 1 and Table 2). 

HDL in NAFLD and ALD was significantly lower compared to the controls, with the lowest values in NAFLD. Triglyceride concentrations were highest in the NAFLD group (Table 1 and Table 2). 

Creatinine was highest in male NAFLD patients (0.99 (0.91, 1.04) vs. 0.78 (0.71, 0.91), *p* = 0.001). Urea nitrogen presented significant differences between all groups in male patients (Table 2). Uric acid was significantly higher in NAFLD than in controls (Table 1 and Table 2). 

Thrombocytes were only significantly decreased in male patients with NAFLD compared to controls and to ALD. C-reactive protein levels in NALFD and ALD were significantly higher than in the controls (Table 1 and Table 2). 

Concerning fibrosis scores, compared to controls, female NAFLD patients had significantly elevated Fib4, FLI scores, and NFS (FiB_Score) scores (Table 1). Males had only an elevated FLI score (Table 2).

### 3.2. Bile Acids 

#### 3.2.1. Females

Compared to healthy controls, female NAFLD patients had significantly increased total (2.89 (1.40, 6.50) vs. 1.18 (0.86, 2.30), *p* = 0.009) and total primary BA (1.62 (1.25, 3.45) vs. 0.65 (0.45, 1.11), *p* = 0.003) concentrations. Women with NAFLD also showed significantly increased total cholic acid (CA, 0.46 (0.21, 0.94) vs. 0.16 (0.08, 0.33), *p* = 0.003), total chenodeoxycholic acid (CDCA, 1.16 (0.68, 2.88) vs. 0.46 (0.33, 0.77), *p* = 0.004), total glycine-conjugated BA (1.89 (1.04, 5.15) vs. 0.65 (0.40, 1.10), *p* = 0.007), and total non-12-OH BA (1.45 (0.80, 3.86) vs. 0.61 (0.45, 1.07) *p* = 0.010, Table 3). Concerning BA subfractions, glycocholic (0.19 (0.12, 0.68) vs. 0.07 (0.04, 0.12), *p* < 0.001), and glycochenodeoxycholic acid (0.84 (0.61, 2.27) vs. 0.25 (0.17, 0.44), *p* = 0.005,) were elevated (Table 3).

#### 3.2.2. Males

Compared to healthy controls, male NAFLD patients had no significantly increased total BA (Table 4). Nevertheless, the BA subfractions glycocholic (0.20 (0.09, 0.35) vs. 0.08 (0.05, 0.16), *p* < 0.001), glycodeoxycholic (0.30 (0.24, 1.11) vs. 0.18 (0.10, 0.41), *p* = 0.003), glycolithocholic (0.014 (0.009, 0.037) vs. 0.007 (0.001, 0.021), *p* = 0.008), lithocholic (0.006 (0.001, 0.019) vs. 0.001 (0.001, 0.001), *p* < 0.001), taurolithocholic (0.0010 (0.0010, 0.0023) vs. 0.0010 (0.0010, 0.0010), *p* < 0.001), and tauroursodeoxycholic acid (0.0010 (0.0010, 0.009) vs. 0.0010 (0.0010, 0.0010), *p* < 0.001) were significantly increased. Cholic acid (0.04 (0.01, 0.12) vs. 0.20 (0.17, 0.28), *p* < 0.001) was significantly decreased (Table 4).

Table 5 provides a brief orientation of the different bile acid profiles of male and female NAFLD patients.

### 3.3. Correlations between Bile Acids and ELF Score

**Figure 1:**
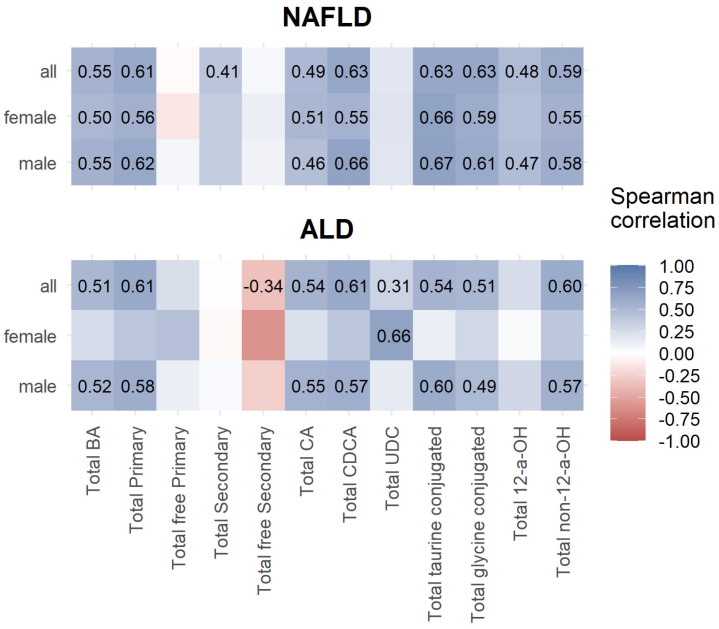
Spearman correlations between ELF score and total bile acids.

Both female and male NAFLD patients had significantly positive correlations between ELF score values and total bile acids, total cholic, total primary, total cholic, total chenodeoxycholic, total taurine-conjugated, total glycine-conjugated, and total non-12-a-OH bile acids (Figure 1). 

In female ALD patients, only total ursodeoxycholic acids showed a significant positive correlation. Male ALD patients had significant positive correlations between total bile acids, total primary, total cholic, total chenodeoxycholic, total taurine-conjugated, total glycine-conjugated, and total non-12-a-OH, bile acids (Figure 1).

**Figure 2:**
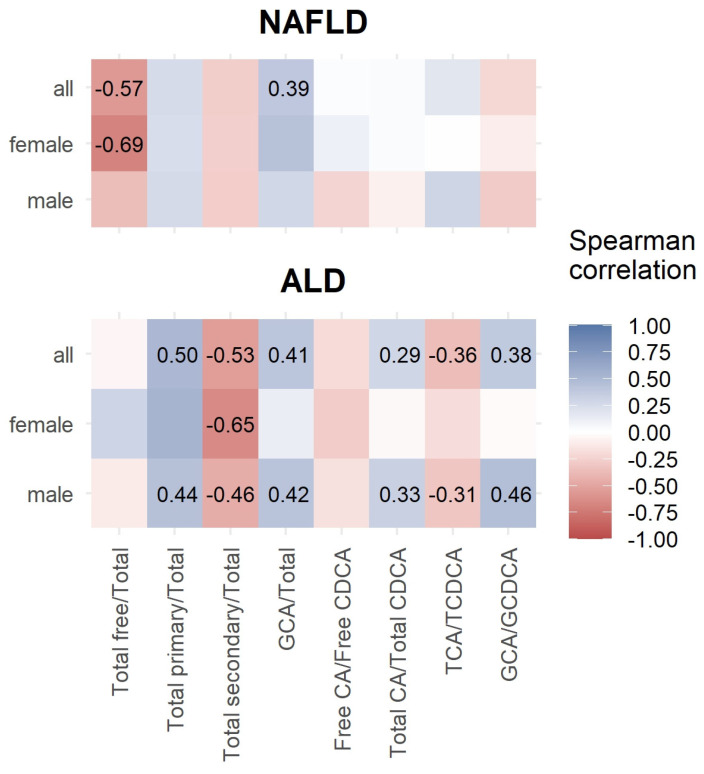
Spearman correlation between ELF score and bile acid ratios.

Concerning bile acid ratios, female NAFLD patients had a significantly negative correlation between total free/total BA and ELF score values. In ALD, females showed no significant correlations. In contrast, males had positively correlating ratios of total primary/total BA, glycocholic acid/total BA, total cholic/total chenodeoxycholic BA, and glycocholic/glycochenodeoxycholic BA with ELF score values. The ratio between taurocholic/taurochenodeoxycholic BA was significantly negatively correlated with ELF score values (Figure 2). 

In NAFLD, total BA, total primary, total secondary, total CA, total CDCA, total taurine-conjugated, total glycine-conjugated, total 12-a-OH, total non-12-a-OH, and ratios of total free/total BA, and GCA/total BA were significantly higher in cases of high ELF scores above 9.8. vs. cases with low/moderate ELF scores lower than 9.8. In AFLD, total UDC BA, was also higher in cases of high ELF scores (Table 6).

### 3.4. Bile Acids in NAFLD without and with NASH

Table 7 shows lab parameters and bile acids in patients with NAFLD without and with NASH. Except in the ELF scores, no significant differences were seen.

## 4. Discussion

We found specific changes in bile acid (BA) concentrations and ratios in NAFLD compared to controls and ALD.

With respect to concentrations of single BA, only GCA and the ratio GCA/total BA showed an outstanding elevation in both men and women.

Female NAFLD patients had significantly elevated levels of total, total primary, total cholic, and total chenodeoxycholic BA. Moreover, a significant absolute increase in total glycine-conjugated BA was evident in female NAFLD. The secondary BA were not significantly different to controls, but higher total primary BA may indicate a gender-dependent disposition to cholestasis [22]. Accordingly [23], the female controls had lower median levels of total primary BA compared to male controls.

Total non-12-a-OH BA were increased in female NAFLD patients, indicating a higher activation of the alternative BA synthesis pathway. This pathway is thought to improve lipid and glucose metabolism and help to detoxify harmful intermediates of BA. The higher activation may represent a healing response, protection against further damage or compensation for the overload of BA [24].

Male NAFLD patients had lower ratios of total free/total BA, free cholic acid/free chenodeoxycholic acid, total cholic acid/total chenodeoxycholic acid, and taurocholic/taurochenodeoxycholic acid. The ratios of glycocholic acid/total BA and glycocholic/glycochenodeoxycholic acid were significantly higher.

Thus, men with fatty liver may have more conjugated primary bile acids than their female counterparts. Male NAFLD patients also had an absolute and relative higher glycine conjugation of CA compared to CDCA, and taurine conjugation of CDCA was relatively higher.

Male NAFLD patients presented with a relative increase in TCDCA, but also tauroursodeoxycholic acid (TUDCA), which decreases ER stress in vitro [17], and the secondary bile acids LCA, GLCA, TLCA, and GDCA were significantly elevated in male NAFLD patients.

Alterations in physiological sexual hormone levels may contribute to fatty liver disease. This may occur in women who received oestrogen receptor antagonists as a breast cancer treatment. Moreover, bisphenol A, a component of plastic, may block oestrogen signalling-associated steatosis [25]. Premenopausal women with age-associated normal oestrogen levels may be protected from severe fibrosis compared to men and postmenopausal women.

The expression and activity of enzymes involved in bile acid metabolism are different between males and females [25]. Enzymes related to detoxification of BA (UDP-glucuronosyltransferase (UGT), sulfotransferase (SULT) 2A1 and nuclear receptors (PXR, CAR)) were suppressed in rats fed with a high-fat–cholesterol (HFC) diet. Male HFC-fed rats showed strong and female only slight affection [26]. However, whether these sex differences are transmittable to humans needs to be confirmed.

The accumulation of BA can be cytotoxic, and an imbalance of BA with their distinct properties can contribute to liver disease. Bile acids with beneficial properties could be lower, and therefore cannot perform their physiological function adequately and BA with specific harmful effects could prevail.

Cholic acid and TCDCA were shown to induce anti-apoptotic proteins and mRNA in hepatocytes [27]. This fact may influence the course of NAFLD by stimulation of cell proliferative processes.

Interestingly, we found no significant differences in the bile acid profiles between NAFLD patients without NASH compared to NAFLD patients with progression of NAFLD to NASH. These results contradict a study by Kasai et al. [28] who found fecal and serum BA and C4 concentrations high in patients with NAFLD with worsening of fibrosis. Possibly, our number of patients with NASH was too low to detect a significant difference.

The diagnostic value of bile acid analysis together with other potential markers for the individual metabolic state of a patient warrants clarification in further studies. Other factors that influence BA composition like the gut microbiome and genetic constellations may be important, but they are still not feasible outside of research. Aspects like circadian rhythm, the chance of short-term changes due to diet, and applicability to other population groups like children may play a role. They may not only be of diagnostic value or help in the exclusion of advanced stages of disease, but it could be possible to find robust biomarkers that give insight into patients’ specific metabolic dysfunction to predict the development of (liver) disease or apply treatment in a very personalised way.

Although there were some similar trends in men and women, our data show that it is necessary to stratify analyses for sex. It would be important to understand the sex difference in physiological bile acid metabolism more in-depth for later use of certain metabolites as diagnostic markers.

It is likely that further markers will add up to existing panels that are already used to increase sensitivity and specificity. A two-phase strategy, which consists of calculating FIB-4 scores first followed by ELF tests to exclude advanced liver damage in the majority of patients has been recently reported to increase detection of liver disease [29].

Besides the impact on the individual suffering from liver disease, the effect on the healthcare system and associated costs cannot be underestimated. Therefore, excluding cases in primary care through cost-efficient and easy-to-perform diagnostic procedures like using a sequence like this would be adequate to lower expenditures [30].

Limitations: The cross-sectional design of our study is a limitation factor. The number of patients with progressive NAFLD (NASH) is too low to discuss bile acid fractions as a factor for NAFLD fibrosis progression. In this study, we do not have any information about the hormone levels or menopausal status of our patients. Direct comparison of our NAFLD and ALD cohorts may be biased by different stages of liver disease. Another limitation is the relatively low number of investigated NAFLD patients.

## 5. Conclusions

NAFLD associates with distinctive BA profiles compared to ALD and healthy controls. Interestingly, female NAFLD patients had a completely different profile compared to men with NAFLD. The only similarity in both sexes was the higher absolute and relative values of glycocholic acid (Table 5).

In female patients, total BA, total primary BA, total CA, glycine-conjugated BA, glycochenodeoxycholic, and total non-12-a-OH acids were significantly elevated. In contrast, men had significant elevations of glycodeoxycholic, glycolithocholic, lithocholic, taurolithocholic, and tauroursodeoxycholic acid, whereas free cholic acid was significantly decreased.

The causes for these gender-related differences remain to be clarified in future studies with a higher number of investigated patients. An elevated number of total non-12-a-OH BA in female NAFLD patients may represent stronger stimulation of the alternative bile acid pathway. This may indicate a gender-specific compensatory reaction of the liver cells to fat storage injury.

Apart from the underlying mechanisms, our data underline that an improved understanding of individual BA profiles is important to better characterise this disease.

## Figures and Tables

**Table 3:** Bile acids of female participants.

Characteristic (µmol/L)	NAFLD, N = 17 ^1^	Controls, N = 52 ^1^	ALD, N = 12 ^1^	NAFLD vs. Controls ^2^	NAFLD vs. ALD ^2^	Controls vs. ALD ^2^
Total BA (µmol/L)	2.89 (1.40, 6.50)	1.18 (0.86, 2.30)	0.95 (0.50, 1.90)	**0.009**	0.049	0.327
Total primary BA	1.62 (1.25, 3.45)	0.65 (0.45, 1.11)	0.49 (0.27, 1.05)	**0.003**	0.049	0.331
Total free primary BA	0.33 (0.13, 0.58)	0.21 (0.11, 0.33)	0.00 (0.00, 0.11)	0.197	**0.002**	**<0.001**
Total secondary BA	1.23 (0.33, 3.58)	0.54 (0.42, 1.07)	0.34 (0.18, 0.68)	0.156	0.035	0.049
Total free secondary BA	0.40 (0.21, 0.48)	0.22 (0.10, 0.32)	0.15 (0.08, 0.25)	0.092	**0.013**	0.164
Total CA	0.46 (0.21, 0.94)	0.16 (0.08, 0.33)	0.07 (0.00, 0.21)	**0.003**	**0.008**	0.070
Total CDCA	1.16 (0.68, 2.88)	0.46 (0.33, 0.77)	0.42 (0.26, 0.89)	**0.004**	0.088	0.655
Total UDC	0.17 (0.07, 0.22)	0.09 (0.05, 0.18)	0.00 (0.00, 0.00)	0.126	**0.004**	**<0.001**
Total taurine-conjugated BA	0.17 (0.10, 0.36)	0.05 (0.03, 0.15)	0.01 (0.01, 0.24)	0.032	0.029	0.028
Total glycine-conjugated BA	1.89 (1.04, 5.15)	0.65 (0.40, 1.10)	0.74 (0.32, 1.25)	**0.007**	0.066	0.923
Total 12-a-OH BA	1.59 (0.46, 3.27)	0.59 (0.43, 1.02)	0.41 (0.19, 0.75)	0.035	0.035	0.109
Total non-12-a-OH BA	1.45 (0.80, 3.86)	0.61 (0.45, 1.07)	0.44 (0.28, 0.93)	**0.010**	0.048	0.244
*Chenodeoxycholic acid*	0.20 (0.09, 0.41)	0.15 (0.07, 0.22)	0.00 (0.00, 0.11)	0.234	0.014	**0.004**
*Cholic acid*	0.06 (0.02, 0.24)	0.03 (0.02, 0.11)	0.00 (0.00, 0.00)	0.350	**<0.001**	**<0.001**
*Deoxycholic acid*	0.20 (0.09, 0.34)	0.17 (0.07, 0.24)	0.12 (0.05, 0.25)	0.372	0.222	0.563
*Glycocholic acid*	0.19 (0.12, 0.68)	0.07 (0.04, 0.12)	0.07 (0.00, 0.17)	**<0.001**	0.025	0.468
*Glycochenodeoxycholic acid*	0.84 (0.61, 2.27)	0.25 (0.17, 0.44)	0.40 (0.20, 0.62)	**0.005**	0.127	0.377
*Glycodeoxycholic acid*	0.77 (0.07, 2.26)	0.24 (0.14, 0.48)	0.04 (0.00, 0.23)	0.103	**0.008**	**0.008**
*Glycolithocholic acid*	0.015 (0.010, 0.026)	0.016 (0.012, 0.026)	0.001 (0.001, 0.001)	0.403	**0.008**	**<0.001**
*Glycoursodeoxycholic acid*	0.08 (0.03, 0.13)	0.04 (0.02, 0.07)	0.00 (0.00, 0.03)	0.061	**0.011**	**0.010**
*Lithocholic acid*	0.003 (0.001, 0.011)	0.007 (0.003, 0.013)	0.001 (0.001, 0.001)	0.330	**<0.001**	**<0.001**
*Taurocholic acid*	0.02 (0.01, 0.09)	0.01 (0.00, 0.03)	0.00 (0.00, 0.03)	0.035	0.048	0.048
*Taurochenodeoxycholic acid*	0.14 (0.04, 0.24)	0.03 (0.02, 0.08)	0.00 (0.00, 0.09)	0.025	0.018	**0.007**
*Taurodeoxycholic acid*	0.01 (0.00, 0.04)	0.01 (0.00, 0.04)	0.00 (0.00, 0.00)	0.954	0.044	**0.010**
*Taurolithocholic acid*	0.0010 (0.0010, 0.0010)	0.0010 (0.0010, 0.0010)	0.0010 (0.0010, 0.0010)	0.740	0.143	0.151
*Tauroursodeoxycholic acid*	0.001 (0.001, 0.008)	0.001 (0.001, 0.001)	0.001 (0.001, 0.001)	0.060	0.268	0.931
*Ursodeoxycholic acid*	0.08 (0.04, 0.14)	0.04 (0.02, 0.09)	0.00 (0.00, 0.00)	0.063	**<0.001**	**<0.001**
Total free/total BA	0.08 (0.02, 0.22)	0.16 (0.09, 0.24)	0.01 (0.00, 0.11)	0.116	0.054	**0.004**
Total primary/total BA	0.59 (0.48, 0.61)	0.51 (0.48, 0.56)	0.60 (0.52, 0.82)	0.153	0.580	0.058
GCA/total BA	0.10 (0.07, 0.13)	0.06 (0.04, 0.09)	0.08 (0.04, 0.09)	**0.015**	0.191	0.813
Free CA/free CDCA	0.4 (0.2, 0.9)	0.4 (0.1, 1.9)	1.0 (0.0, 1.0)	0.899	0.964	0.489
Total CA/total CDCA	0.38 (0.28, 0.57)	0.34 (0.20, 0.50)	0.17 (0.06, 0.41)	0.453	0.044	0.043
TCA/TCDCA	0.26 (0.21, 0.40)	0.17 (0.09, 0.29)	1.00 (0.49, 1.00)	0.036	**0.001**	**<0.001**
GCA/GCDCA	0.36 (0.21, 0.62)	0.27 (0.21, 0.39)	0.17 (0.06, 0.40)	0.257	0.054	0.065

^1^ Median (IQR), bile acid subtypes shown in italic letters. ^2^ The significance level is 0.0167 due to Bonferroni correction (shown in bold *p* values).

**Table 4:** Bile acids for male participants.

Characteristic (µmol/L)	NAFLD, N = 28 ^1^	Controls, N = 51 ^1^	ALD, N = 45 ^1^	NAFLD vs. Controls ^2^	NAFLD vs. ALD ^2^	Controls vs. ALD ^2^
Total BA	2.88 (1.49, 3.97)	1.85 (1.30, 2.43)	1.50 (0.80, 2.90)	0.090	**0.012**	0.129
Total primary BA	1.23 (0.68, 2.61)	0.97 (0.64, 1.50)	1.06 (0.43, 1.88)	0.247	0.158	0.658
Total free primary BA	0.32 (0.18, 0.54)	0.32 (0.24, 0.54)	0.05 (0.00, 0.24)	0.416	**<0.001**	**<0.001**
Total secondary BA	1.12 (0.75, 1.90)	0.82 (0.59, 1.19)	0.46 (0.20, 0.76)	0.047	**<0.001**	**<0.001**
Total free secondary BA	0.47 (0.33, 0.67)	0.41 (0.27, 0.56)	0.18 (0.04, 0.32)	0.587	**<0.001**	**<0.001**
Total CA	0.34 (0.15, 0.67)	0.38 (0.27, 0.51)	0.14 (0.06, 0.30)	0.718	**0.005**	**<0.001**
Total CDCA	0.83 (0.55, 2.07)	0.59 (0.44, 0.98)	0.89 (0.36, 1.56)	0.049	0.424	0.398
Total UDC	0.17 (0.08, 0.50)	0.20 (0.20, 0.30)	0.00 (0.00, 0.20)	0.376	**<0.001**	**<0.001**
Total taurine-conjugated BA	0.10 (0.05, 0.50)	0.16 (0.10, 0.22)	0.05 (0.01, 0.17)	0.884	**0.007**	**0.001**
Total glycine-conjugated BA	1.01 (0.67, 2.72)	0.88 (0.42, 1.28)	1.00 (0.52, 1.99)	0.071	0.202	0.440
Total 12-a-OH BA	1.15 (0.64, 2.47)	0.93 (0.63, 1.34)	0.49 (0.26, 0.81)	0.299	**<0.001**	**<0.001**
Total non-12-a-OH BA	1.36 (0.81, 2.27)	0.88 (0.63, 1.25)	0.98 (0.37, 2.07)	0.028	0.124	0.942
*Chenodeoxycholic acid*	0.27 (0.13, 0.40)	0.14 (0.07, 0.32)	0.04 (0.00, 0.22)	0.065	**<0.001**	**<0.001**
*Cholic acid*	0.04 (0.01, 0.12)	0.20 (0.17, 0.28)	0.00 (0.00, 0.00)	**<0.001**	**<0.001**	**<0.001**
*Deoxycholic acid*	0.27 (0.13, 0.40)	0.26 (0.15, 0.43)	0.13 (0.00, 0.28)	0.743	**0.009**	**<0.001**
*Glycocholic acid*	0.20 (0.09, 0.35)	0.08 (0.05, 0.16)	0.14 (0.05, 0.25)	**<0.001**	0.037	0.299
*Glycochenodeoxycholic acid*	0.42 (0.25, 1.26)	0.40 (0.23, 0.69)	0.75 (0.29, 1.19)	0.401	0.679	0.034
*Glycodeoxycholic acid*	0.30 (0.24, 1.11)	0.18 (0.10, 0.41)	0.08 (0.00, 0.23)	**0.003**	**<0.001**	**0.003**
*Glycolithocholic acid*	0.014 (0.009, 0.037)	0.007 (0.001, 0.021)	0.001 (0.001, 0.001)	**0.008**	**<0.001**	**<0.001**
*Glycoursodeoxycholic acid*	0.07 (0.03, 0.19)	0.05 (0.03, 0.12)	0.00 (0.00, 0.15)	0.690	0.022	0.033
*Lithocholic acid*	0.006 (0.001, 0.019)	0.001 (0.001, 0.001)	0.001 (0.001, 0.001)	**<0.001**	**<0.001**	**<0.001**
*Taurocholic acid*	0.02 (0.01, 0.11)	0.06 (0.04, 0.07)	0.00 (0.00, 0.00)	0.185	**<0.001**	**<0.001**
*Taurochenodeoxycholic acid*	0.08 (0.03, 0.29)	0.05 (0.03, 0.07)	0.03 (0.00, 0.10)	0.060	0.027	0.346
*Taurodeoxycholic acid*	0.02 (0.01, 0.05)	0.04 (0.03, 0.07)	0.00 (0.00, 0.03)	0.080	**<0.001**	<0.001
*Taurolithocholic acid*	0.0010 (0.0010, 0.0023)	0.0010 (0.0010, 0.0010)	0.0010 (0.0010, 0.0010)	**<0.001**	**<0.001**	>0.999
*Tauroursodeoxycholic acid*	0.001 (0.001, 0.009)	0.001 (0.001, 0.001)	0.001 (0.001, 0.001)	**<0.001**	**<0.001**	0.123
*Ursodeoxycholic acid*	0.07 (0.02, 0.31)	0.16 (0.12, 0.19)	0.00 (0.00, 0.00)	0.274	**<0.001**	**<0.001**
Total free/total BA	0.13 (0.07, 0.20)	0.20 (0.14, 0.30)	0.04 (0.00, 0.10)	**0.009**	**<0.001**	**<0.001**
Total primary/total BA	0.52 (0.47, 0.59)	0.57 (0.50, 0.63)	0.68 (0.53, 0.85)	0.210	**0.003**	**0.003**
GCA/total BA	0.09 (0.06, 0.11)	0.04 (0.03, 0.08)	0.07 (0.04, 0.13)	**<0.001**	0.392	0.048
Free CA/free CDCA	0.18 (0.08, 0.47)	1.68 (0.88, 3.22)	0.17 (0.01, 1.00)	**<0.001**	0.643	**<0.001**
Total CA/total CDCA	0.33 (0.24, 0.40)	0.64 (0.43, 0.86)	0.14 (0.08, 0.28)	**<0.001**	**<0.001**	**<0.001**
TCA/TCDCA	0.30 (0.20, 0.40)	1.08 (0.56, 1.58)	0.31 (0.03, 1.00)	**<0.001**	0.855	**<0.001**
GCA/GCDCA	0.37 (0.30, 0.60)	0.24 (0.16, 0.32)	0.16 (0.10, 0.27)	**<0.001**	**<0.001**	**0.009**

^1^ Median (IQR), bile acid subtypes marked in italic letters. ^2^ The significance level is 0.0167 due to Bonferroni correction (shown in bold *p* values).

**Table 5:** Overview of changes in bile acids (arrow up/down for comparison NAFLD vs. controls).

	Significant Different BA Profiles in NAFLD vs. Controls	
	Total Bile Acids	Individual Bile Acids
Female NAFLD	↑ total BA * ↑ total primary BA ** ↑ total CA ** ↑ total CDCA * ↑ total glycine-conjugated BA * ↑ total non-12-a-OH BA *	↑ Glycocholic acid *** ↑ Glycochenodeoxycholic acid *
Male NAFLD		↓ Cholic acid *** ↑ Glycocholic acid *** ↑ Glycodeoxycholic acid ** ↑ Glycolithocholic acid * ↑ Lithocholic acid *** ↑ Taurolithocholic acid *** ↑ Tauroursodeoxycholic acid ***

* for *p* < 0.0167, ** for *p* < 0.0033, and *** for *p* < 0.00033.

**Table 6:** Routine lab parameters and bile acids in patients with elevated ELF score values. Low/moderate = ELF score < 9.8, high = ELF score ≥ 9.8.

	NAFLD, N = 41			ALD, N = 54		
Characteristic	Low/Moderate, N = 27 ^1^	High, N = 14 ^1^	*p*-Value ^2^	Low/Moderate, N = 38 ^1^	High, N = 16 ^1^	*p*-Value ^2^
Gender			0.142			0.474
female	9 (33.3%)	8 (57.1%)		9 (23.7%)	2 (12.5%)	
male	18 (66.7%)	6 (42.9%)		29 (76.3%)	14 (87.5%)	
Age	45 (14)	60 (8)	**<0.001**	48 (9)	48 (10)	0.948
C-reactive protein (mg/dL)	1.5 (0.8, 4.1)	2.2 (0.6, 6.4)	0.674	2 (1, 4)	4 (3, 15)	**0.010**
ALT (U/L)	81 (50, 126)	77 (59, 150)	0.891	28 (22, 42)	37 (28, 62)	0.229
total BA	2.1 (1.5, 3.0)	7.2 (2.9, 9.5)	**0.004**	1.1 (0.6, 1.7)	3.3 (1.6, 6.9)	**<0.001**
Total Primary	1.18 (0.66, 1.78)	3.70 (1.61, 5.66)	**0.001**	0.6 (0.2, 1.4)	2.3 (1.4, 6.6)	**<0.001**
Total free Primary	0.36 (0.19, 0.57)	0.26 (0.12, 0.56)	0.357	0.04 (0.00, 0.14)	0.08 (0.00, 0.49)	0.312
Total Secondary	0.87 (0.70, 1.26)	3.53 (0.54, 4.41)	**0.026**	0.40 (0.18, 0.65)	0.52 (0.30, 1.22)	0.169
Total free Secondary	0.44 (0.25, 0.57)	0.47 (0.32, 0.61)	0.525	0.23 (0.07, 0.32)	0.12 (0.03, 0.20)	0.223
Total CA	0.24 (0.16, 0.54)	0.81 (0.25, 1.56)	**0.025**	0.08 (0.01, 0.18)	0.53 (0.17, 1.70)	**<0.001**
Total CDCA	0.68 (0.50, 1.15)	2.99 (1.37, 4.10)	**<0.001**	0.48 (0.22, 1.13)	1.97 (1.10, 4.88)	**<0.001**
Total UDC	0.15 (0.06, 0.34)	0.24 (0.10, 0.74)	0.283	0.00 (0.00, 0.10)	0.10 (0.00, 0.50)	**0.029**
Total taurine conjugated	0.09 (0.05, 0.17)	0.44 (0.20, 1.32)	**0.001**	0.01 (0.01, 0.07)	0.30 (0.06, 1.86)	**<0.001**
Total glycine conjugated	0.85 (0.66, 1.81)	5.40 (2.11, 7.30)	**<0.001**	0.66 (0.31, 1.36)	1.97 (1.04, 5.02)	**<0.001**
Total 12-a-OH	0.97 (0.60, 1.60)	3.51 (0.92, 5.08)	**0.013**	0.41 (0.21, 0.60)	0.79 (0.43, 2.61)	**0.008**
Total non-12-a-OH	1.07 (0.78, 1.53)	3.78 (1.69, 4.61)	**0.002**	0.50 (0.28, 1.20)	2.11 (1.18, 5.19)	**<0.001**
total free/Total	0.18 (0.10, 0.32)	0.07 (0.01, 0.08)	**<0.001**	0.04 (0.00, 0.12)	0.02 (0.00, 0.06)	0.289
total primary/total BA	0.52 (0.44, 0.59)	0.57 (0.48, 0.60)	0.402	0.63 (0.47, 0.80)	0.76 (0.68, 0.93)	**0.019**
Total secondary/Total	0.48 (0.41, 0.56)	0.43 (0.40, 0.53)	0.355	0.39 (0.21, 0.58)	0.27 (0.07, 0.35)	**0.011**
GCA/Total	0.08 (0.05, 0.10)	0.11 (0.09, 0.12)	**0.033**	0.06 (0.01, 0.09)	0.12 (0.07, 0.13)	**0.003**
Free CA/Free CDCA	0.2 (0.1, 0.6)	0.3 (0.0, 0.8)	0.670	0.59 (0.01, 1.00)	0.06 (0.01, 1.00)	0.404
Total CA/Total CDCA	0.34 (0.28, 0.51)	0.32 (0.22, 0.39)	0.501	0.11 (0.04, 0.20)	0.27 (0.14, 0.40)	**0.009**
T-CA/T-CDCA	0.28 (0.18, 0.39)	0.28 (0.22, 0.37)	0.837	1.00 (0.05, 1.00)	0.27 (0.03, 0.43)	**0.012**
G-CA/G-CDCA	0.37 (0.27, 0.64)	0.35 (0.22, 0.37)	0.182	0.12 (0.01, 0.21)	0.26 (0.17, 0.35)	**0.003**

^1^ *n* (%); mean (SD); median (IQR). ^2^ The significance level is 0.0167 due to Bonferroni correction (shown in bold *p* values).

**Table 7:** Lab parameters and bile acids in patients with NAFLD without and with NASH.

Characteristic	NAFLD without NASH, N = 24 ^1^	NAFLD with NASH, N = 20 ^1^	*p*-Value ^2^
Gender			0.429
female	8 (33.3%)	9 (45.0%)	
male	16 (66.7%)	11 (55.0%)	
Age	48 (15)	56 (11)	0.050
C-reactive protein (mg/dL)	1.7 (0.8, 3.7)	2.2 (1.1, 6.2)	0.425
ALT (U/L)	72 (51, 91)	97 (57, 155)	0.207
ELF score cut			0.026
low/moderate	17 (81.0%)	9 (47.4%)	
high	4 (19.0%)	10 (52.6%)	
total BA	3.0 (1.4, 5.1)	2.9 (1.9, 6.4)	0.768
Total primary	1.41 (0.65, 2.69)	1.78 (1.10, 3.45)	0.444
Total free primary	0.27 (0.12, 0.52)	0.45 (0.24, 0.65)	0.225
Total secondary	1.13 (0.70, 2.44)	1.21 (0.61, 2.45)	0.760
Total free secondary	0.46 (0.21, 0.66)	0.44 (0.33, 0.50)	0.854
Total CA	0.29 (0.14, 1.06)	0.48 (0.22, 0.63)	0.768
Total CDCA	0.83 (0.47, 2.03)	1.15 (0.64, 2.88)	0.352
Total UDC	0.17 (0.07, 0.67)	0.15 (0.07, 0.41)	0.637
Total taurine conjugated	0.13 (0.05, 0.42)	0.18 (0.09, 0.49)	0.390
Total glycine conjugated	1.4 (0.7, 3.3)	1.8 (0.8, 4.2)	0.612
Total 12-a-OH	1.32 (0.61, 2.59)	1.29 (0.81, 3.24)	0.897
Total non-12-a-OH	1.42 (0.73, 2.59)	1.31 (0.96, 2.91)	0.687
Total free/total	0.14 (0.05, 0.22)	0.12 (0.07, 0.18)	0.823
Total primary/total	0.52 (0.46, 0.61)	0.57 (0.52, 0.60)	0.430
GCA/total	0.09 (0.05, 0.12)	0.09 (0.08, 0.11)	0.805
Free CA/free CDCA	0.3 (0.1, 1.2)	0.2 (0.1, 0.5)	0.207
Total CA/total CDCA	0.35 (0.24, 0.52)	0.32 (0.24, 0.42)	0.663
TCA/TCDCA	0.30 (0.20, 0.40)	0.30 (0.24, 0.43)	0.733
GCA/GCDCA	0.39 (0.31, 0.66)	0.34 (0.21, 0.55)	0.234

^1^ n (%); mean (SD); median (IQR). ^2^ The significance level is 0.0167 due to Bonferroni correction (shown in bold *p* values).

## Data Availability

All data are available from the corresponding author.
